# Distinct Profiles and New Pharmacological Targets for Heart Failure with Preserved Ejection Fraction

**DOI:** 10.31083/j.rcm2507270

**Published:** 2024-07-23

**Authors:** Alberto Palazzuoli, Paolo Severino, Andrea D’Amato, Vincenzo Myftari, Lucia Tricarico, Michele Correale, Giuseppe Dattilo, Francesco Fioretti, Savina Nodari

**Affiliations:** ^1^Cardiovascular Diseases Unit, Cardio Thoracic and Vascular Department Le Scotte Hospital University of Siena, 53100 Siena, Italy; ^2^Department of Clinical, Internal Anesthesiological and Cardiovascular Sciences University La Sapienza Rome, 00161 Roma, Italy; ^3^UO Cardiologia Universitaria – Intensive Coronary Unit Policlinico Riuniti Foggia, 71122 Foggia, Italy; ^4^Cardiology Unit, Department of Clinical and Experimental Medicine, University of Messina, 98122 Messina, Italy; ^5^Cardiology Section, Department of Medical and Surgical Specialties, Radiological Sciences and Public Health, Spedali Civili Hospital and University of Brescia, 25123 Brescia, Italy

**Keywords:** HFpEF phenotype, treatment, SGLT2i, ARNIs, MRA, GLP-1

## Abstract

**Background:**

Heart failure with preserved ejection fraction (HFpEF) is a multifactorial condition with a variety of pathophysiological causes and morphological manifestations. The inclusion criteria and patient classification have become overly simplistic due to the customary differentiation regarding the ejection fraction (EF) cutoff. EF is considered a measure of systolic function; nevertheless, it only represents a portion of the true contractile state and has been shown to have certain limits due to methodological and hemodynamic irregularities.

**Methods:**

As a result, broader randomized clinical trials have yet to incorporate the most recent criteria for HFpEF diagnosis, leading to a lack of data consistency and confusion in interpreting the results. The primary variations between the bigger clinical trials published in this context concerning patient selection and echocardiographic characteristics were analyzed. For all these reasons, we aim to clarify the main features and clinical impact of HFpEF in a study combining imaging, bio-humoral analysis, and clinical history to identify the specific subgroups that respond better to tailored treatment.

**Results:**

Disparate clinical characteristics and a lack of uniform diagnostic standards may cause suboptimal therapeutic feedback. To optimize treatment, we suggest shifting the paradigm from the straightforward EF measurement to a more comprehensive model that considers additional information, such as structural traits, related disorders, and biological and environmental data. Therefore, by evaluating certain echocardiographic and clinical factors, a stepwise diagnostic procedure may be useful in identifying patients at high risk, subjects with early HFpEF, and those with evident HFpEF.

**Conclusions:**

The present assessment underscores the significance of the precision medicine approach in guaranteeing optimal patient outcomes by providing the best care according to each distinct profile.

## 1. Introduction

Heart failure with preserved ejection fraction (HFpEF) affects around half of all heart failure (HF) cases, and throughout the past 20 years, this percentage has been rising [1, 2]. HFpEF prevalence in the adult population is around 1–3%, although it is increasing in the general global population [3]. Additionally, the 1-year mortality is about 6.3%, reaching 75.7% at 5 years, which is comparable to heart failure with reduced ejection fraction (HFrEF). While cardiovascular death remains the primary outcome for HF, non-cardiovascular causes are increasingly significant in HFpEF [4, 5, 6].

Compared to HF with impaired systolic function, HFpEF is regarded as a distinct entity with specific molecular vascular myocardial cell adaptations and a detailed pathophysiological profile (HFrEF) [7]. This evaluation is based on certain presumptions regarding the anatomy of the left ventricle (LV), the composition and structure of the heart, the geometry of the intrinsic fiber myocytes, the thickness of the vessels, and the effects of therapy [8]. Consequently, it is a diverse syndrome with a high comorbidity burden characterized by several related diseases and different clinical phenotypes. The considerable variety of enrollment criteria in the larger multicenter trials showed a lack of diagnostic consistency, and criteria uniformity is required for both the time course evaluation and the adopted treatment [9]. Understanding the underlying pathophysiological processes that generate hemodynamic changes and cardiovascular dysfunctions is crucial for addressing the optimized therapy according to different phenotypes. For example, patients with HFpEF and a prevalent coronary microvascular impairment as the main pathophysiological mechanism may benefit more from drugs acting on microcirculation that reduce coronary resistance, improving coronary blood flow. Alternatively, patients with HFpEF and prevalent extracellular fibrosis may benefit more from drugs with anti-fibrotic properties, which reduce collagen extracellular storage. Patients with early supraventricular tachyarrhythmias (SVTs) may benefit from early ablation procedures because recurrent supraventricular tachyarrhythmia (SVT) may trigger electrical disorder persistence and HFpEF progression. In conclusion, the emerging role of genetics and epigenetics may open new perspectives in HFpEF classification and treatment. The contrasting results of novel and conventional treatments emphasize the need to search for a more suitable mechanistic strategy based on more customized therapy that incorporates data on lifestyle, genetic, biochemical, environmental, and cardiovascular (CV) risk factors [10]. This review aims to revise the knowledge concerning this complex syndrome, particularly regarding the different phenotypes and the current and potential therapeutic targets in HFpEF.

## 2. Evolution of Classification and Diagnostic Innovations in HFpEF

### 2.1 Rising Awareness and Understanding of HFpEF

The understanding of HFpEF has transformed from a relatively overlooked condition to one now receiving growing recognition. HFpEF is a clinical syndrome distinguished by the manifestation of HF signs and symptoms due to augmented LV filling pressure, even in the presence of a normal or near-normal (≥50%) LV ejection fraction (LVEF) [11]. It is also characterized by a marked increase in natriuretic peptides (NPs), the presence of left ventricular hypertrophy (LVH), and/or left atrial (LA) enlargement, along with some level of diastolic dysfunction. For this reason, it was frequently considered a diagnosis of exclusion. However, the absence of well-defined criteria posed difficulties in both research and clinical contexts; thus, it became evident that the syndrome is heterogeneous, involving various comorbidities and contributing factors [12]. As a clinical syndrome, the delineation of HFpEF is complex, mainly due to the considerable variability in the clinical manifestations of patients along several dimensions: multiple comorbidities can alter clinical symptoms and signs; it also involves various organ systems, encompassing both cardiac and non-cardiac expressions [13, 14].

The increasing knowledge about HFpEF syndrome and the complexity of this condition are responsible for the evolutive classification of the HFpEF concept over the previous decade [15]. Until recently, HFpEF was a diagnosis of exclusion, while the term HFpEF implies a definition based solely on LVEF values, which is a rough and simplistic parameter not always associated with the pathophysiological background [16, 17]. However, over the recent years, novel modalities of classification have been introduced, which involve several aspects of the disease, including clinical, imaging, laboratory, metabolomic, and epigenetic parameters. To unravel this complex syndrome, it is necessary to increase the complexity of the classification to distinguish the diagnostic and therapeutic pathways [11, 15, 16].

In this regard, Fayol *et al*. [18] emphasized the role of etiology in HFpEF, demonstrating its usefulness in deciphering HFpEF heterogeneity better. They stratified 2180 patients with HFpEF according to etiology, identifying three main phylogroups. In particular, the best prognosis was observed in patients with idiopathic HFpEF, a group characterized by a high rate of non-cardiac comorbidities, compared to patients with secondary HFpEF due to myocardial hemodynamic loading abnormalities. On the other hand, Shah *et al*. [19] stratified patients according to prognosis to identify useful predictors. Patients with the best prognosis were relatively young, with mild diastolic dysfunction and low natriuretic peptide (NP) levels. Patients with “metabolic HF” were obese with LA dilatation, LVH, and diastolic dysfunction. This group was found to have a risk of death four times higher than the first. The third group included patients with a higher incidence of atrial fibrillation (AF), the highest NP levels, and the most severe diastolic dysfunction, and they were at the highest risk of death.

A different approach was used by Cohen *et al*. [20], who performed a secondary analysis on the Treatment of Preserved Cardiac Function Heart Failure with an Aldosterone Antagonist (TOPCAT) trial, dividing patients according to vascular and cardiac remodeling. The first subgroup included patients with mild LVH and chronic pulmonary hypertension with normal vascular stiffness and a rise in the expression of metalloproteinase; the second subgroup was characterized by older patients with multiple comorbidities, reduced vascular compliance, parietal hypertrophy, and tissue calcification; the third subgroup included obese patients with several metabolic alterations, an increase in renin-angiotensin-aldosterone system mediators levels, and an alteration in lipidic profile and tissue inflammation. The latter group showed a better response to the mineralocorticoid receptor antagonists, demonstrating that the response to therapy in the broad spectrum of HFpEF varies according to the subgroup considered. Moreover, Kyodo *et al*. [21] found that male patients older than 70 years with atherosclerotic vascular disease kidney and heart organ damage had a worse prognosis compared to other HFpEF patients. Beyond the clinical, etiological, and imaging parameters, a new idea of HFpEF classification has integrated several circulating biomarkers, markers of metabolic dysfunction, and microRNAs, which may be useful for diagnosis, prognosis, and cutting-edge personalized therapy [22]. The main circulating biomarkers identified have been related to myocardial injury (i.e., NPs, cardiac troponins), extracellular fibrosis (i.e., ST2, galectin-3, metalloproteinases), inflammation markers (i.e., interleukin-6 (IL-6), pentraxin, tumor necrosis factor-alfa (TNF-alfa)), and markers of endothelial dysfunction (i.e., endothelin-1, vascular cell adhesion molecule (VCAM)). From a metabolomic point of view, impairment of several pathways was identified. In particular, the role of lipids, energy, inflammation, endothelial impairment, and increased collagen synthesis has been shown [22].

### 2.2 Innovations in HFpEF Diagnosis and Classification

Diagnosing HFpEF is based on integrating clinical, imaging, and laboratory parameters [11]. Echocardiography is key in assessing HFpEF beyond estimating the LV diameters, volumes, and LVEF. Echocardiography allows the comprehensive evaluation of left ventricular diastolic function, a hallmark feature of HFpEF. Parameters such as early diastolic mitral inflow velocity (E), late diastolic mitral inflow velocity (A), and the E/A ratio provide information about the diastolic filling pattern. In addition, tissue Doppler imaging enables the assessment of myocardial velocities during diastole, offering insights into LV relaxation [23]. The spironolactone improved diastolic function (E/e’) ratio, which relates mitral inflow velocity to early diastolic tissue velocity, is a valuable parameter indicating elevated left ventricular filling pressures when the ratio exceeds 14. As the diastolic dysfunction progresses, there is a decrease in LV compliance during the atrial contraction phase, which is associated with impaired LV relaxation [24]. Moreover, it has been shown that reduced LV compliance is one of the strongest predictors of less favorable outcomes [25]. The diminished LV compliance leads to an increase in mean LA pressure and dimensions. Echocardiography is the gold standard in the evaluation of the LA volume, and it ought to be assessed after the systolic phase of the LV. Commonly, maximal LA volume is used with high prognostic power [24]; however, minimal LA volume has emerged as a viable alternative in identifying patients who are more at risk of CV events [26, 27]. Beyond the traditional evaluation of the LA volume, the resting LA strain has earned growing importance in the diastolic and HFpEF analysis [28]. Biomarkers have gained prominence in HFpEF diagnosis. NPs are cardiac hormones with cardioprotective properties, secreted by cardiomyocytes in response to pressure or volume overload and neuroendocrine–immune system stimulation. They have been extensively studied as diagnostic and prognostic factors for the disease, and the thresholds have been defined by the last European Society of Cardiology (ESC) guidelines to exclude those unlike HF [11, 29]. Integrating N-terminal pro-B-type natriuretic peptide (NT-proBNP) into the diagnostic algorithm, along with clinical evaluation and imaging studies, holds promise for improving the precision of HFpEF diagnoses and patient care optimization. A recent meta-analysis of 51 studies revealed that NPs demonstrate reliable diagnostic accuracy for identifying HFpEF in non-acute scenarios [30]. However, it is essential to underline different situations that interfere with NP levels, notably with NT-proBNP: age, gender, weight, genetic polymorphisms, renal insufficiency, and arrhythmia (AF in particular) [31].

The underlying myocardial changes in HFpEF still need to be defined. Myocardial histopathological features have been characterized by invasive methods such as endomyocardial biopsy, but cardiac magnetic resonance (CMR) or other imaging techniques can identify them more easily and non-invasively [32]. Over the years, several studies have investigated CMR-derived diastolic functional indices, including transmitral and pulmonary venous velocities, LV and LA strain using myocardial tagging, and, more recently, feature tracking [33]. Additionally, CMR provides early markers for detecting myocardial disease using tissue characterization imaging, which may improve the diagnosis and treatment. Myocardial fibrosis, hypertrophy of cardiomyocytes, coronary microvascular dysfunction (CMD), and inflammation have been recognized as key pathological processes impacting the myocardium in HFpEF [32]. Among CMR parameters, various studies have shown that global longitudinal strain (GLS) and extracellular volume (ECV) can completely discriminate the different phenotypes of HFpEF, with ECV appearing to be the strongest imaging marker that can distinguish hypertension from HFpEF independently [34]. The importance of CMR in HFpEF also lies in its ability to predict patients’ prognosis and outcome. Quantification of myocardial focal fibrosis by late gadolinium enhancement (LGE) may be useful for risk stratification in HFpEF patients, as a larger LGE was significantly associated with a high rate of future CV death and hospitalization for HF [35] (Table 1, Ref. [16, 17, 26, 27, 28, 29, 30, 31, 32]).

**Table 1.** **Summary evolution of the diagnostic framework of HFpEF**.

HFpEF diagnosis	Evolution summary	Reference
Biomarkers integration	The integration of circulating biomarkers, markers of metabolic dysfunction, and microRNAs has been incorporated into HFpEF classification. Identified biomarkers include those related to myocardial injury, extracellular fibrosis, inflammation, and endothelial dysfunction, providing valuable information for diagnosis, prognosis, and personalized therapy.	[16]
Advancements in imaging techniques	Echocardiography assesses diastolic function, while CMR-derived indices, including GLS and ECV, discriminate between different HFpEF phenotypes. LGE on CMR aids in risk stratification.	[17, 26, 27, 28, 29]
Scoring systems and challenges	H2FPEF score complements traditional diagnostic tools. Future research should focus on prospective validation and integrating these scores into routine clinical practice.	[30, 31, 32]
Diagnostic algorithm development	Development of stepwise diagnostic algorithms such as HFA-PEFF, incorporating pre-assessment, echocardiography, functional testing, and final etiology phases. The H2FPEF score provides a practical tool for initial risk stratification.	[30, 31, 32]

HFpEF, heart failure with preserved ejection fraction; CMR, cardiovascular magnetic resonance; LGE, late gadolinium enhancement; GLS, global longitudinal strain; ECV, extracellular volume; H2FPEF, heart 2 preserved ejection fraction; HFA-PEFF, Heart Failure Association-preserved ejection fraction.

Thus, the diagnostic approach can be complex, requiring a global assessment beyond the single echocardiographic and serum parameters. A stepwise algorithm is recommended for the diagnosis of HFpEF and its severity. Several scores have been developed, incorporating common parameters in this pathology [36]. The consensus recommendation of the Heart Failure Association (HFA) of the ESC has developed the Heart Failure Association-preserved ejection fraction (HFA-PEFF) diagnostic algorithm, which consists of four different phases: Pre-assessment (P), echocardiography (E), functional testing (F1), and final etiology (F2). Pre-assessment is typically performed in the ambulatory setting and includes the clinical evaluation of HF symptoms and signs, typical clinical morbidities, and diagnostic laboratory tests aiming to identify the risk of HFpEF (low, intermediate, or high) based on the patient’s risk factors. Echocardiography, as mentioned, provides strong evidence of diastolic dysfunction and high LV filling pressure, which the NT-proBNP measurement must accompany. When ambiguous results are reached in the first steps, an additional stress test or an invasive hemodynamic study is needed to unmask the most challenging presentations [37]. Furthermore, the heart 2 preserved ejection fraction (H2FPEF) score represents a clinical tool that incorporates five easily assessable parameters: Hypertension, AF, age ≥60 years, obesity, and E/e’ ratio >9. The simplicity of this score makes it practical for initial risk stratification in patients suspected of HFpEF, enabling clinicians to identify individuals who may benefit from further diagnostic evaluation [38]. These scores complement traditional diagnostic tools, offering an additional layer of assessment that is particularly valuable in cases with unclear clinical displays. While scoring systems provide valuable contributions, challenges include potential variations in patient populations and the need for continuous refinement. Future research should focus on prospective validation of these scores and their integration into routine clinical practice. Additionally, ongoing efforts to identify and incorporate novel biomarkers and advanced imaging modalities may further enhance the accuracy of diagnostic scoring systems.

The field of HFpEF classification and diagnosis continues to evolve, with ongoing research exploring genetic and molecular factors and the potential role of artificial intelligence in refining diagnostic accuracy [21]. Multimodal approaches integrating clinical, imaging, and biomarker data hold promise for a comprehensive understanding of HFpEF, paving the way for personalized and targeted therapeutic strategies.

## 3. Different HFpEF Phenotypes

Many questions are currently being raised regarding the influence of common cardiovascular risk factors in the general population that can induce HFpEF, the role that comorbidities play in the development of this syndrome, and the precise correlation between this syndrome and underlying cardiac dysfunction. These questions result from the inconsistent findings of clinical trials and the generation of different hypotheses. More recently, some epidemiological studies have proposed that HFpEF represents a terminal phase of various diseases with diverse clinical courses, outcomes, and phenotypes. Distinct patterns and HFpEF subtypes have been revealed by certain cluster analyses of big randomized clinical trials (RCTs). For example, in the TOPCAT trial, latent class analysis identified three primary categories based on left ventricular geometry, clinical profile, risk factor burden, and vascular characteristics [20]. The authors identified three main phenogroups: Group 1 with low NP levels, normal LV geometry, low arterial stiffness, and cardiac events; Group 2 was characterized by high NP levels, increased vascular stiffness associated with LV concentric hypertrophy, LA dilatation and an elevated incidence of cardiovascular events; Group 3 is the typical metabolic pattern with an elevated level of rennin, an abnormal glycemic and lipid profile, an increased inflammatory pattern and a better response to spironolactone treatment [20, 21].

**Fig. 1.**
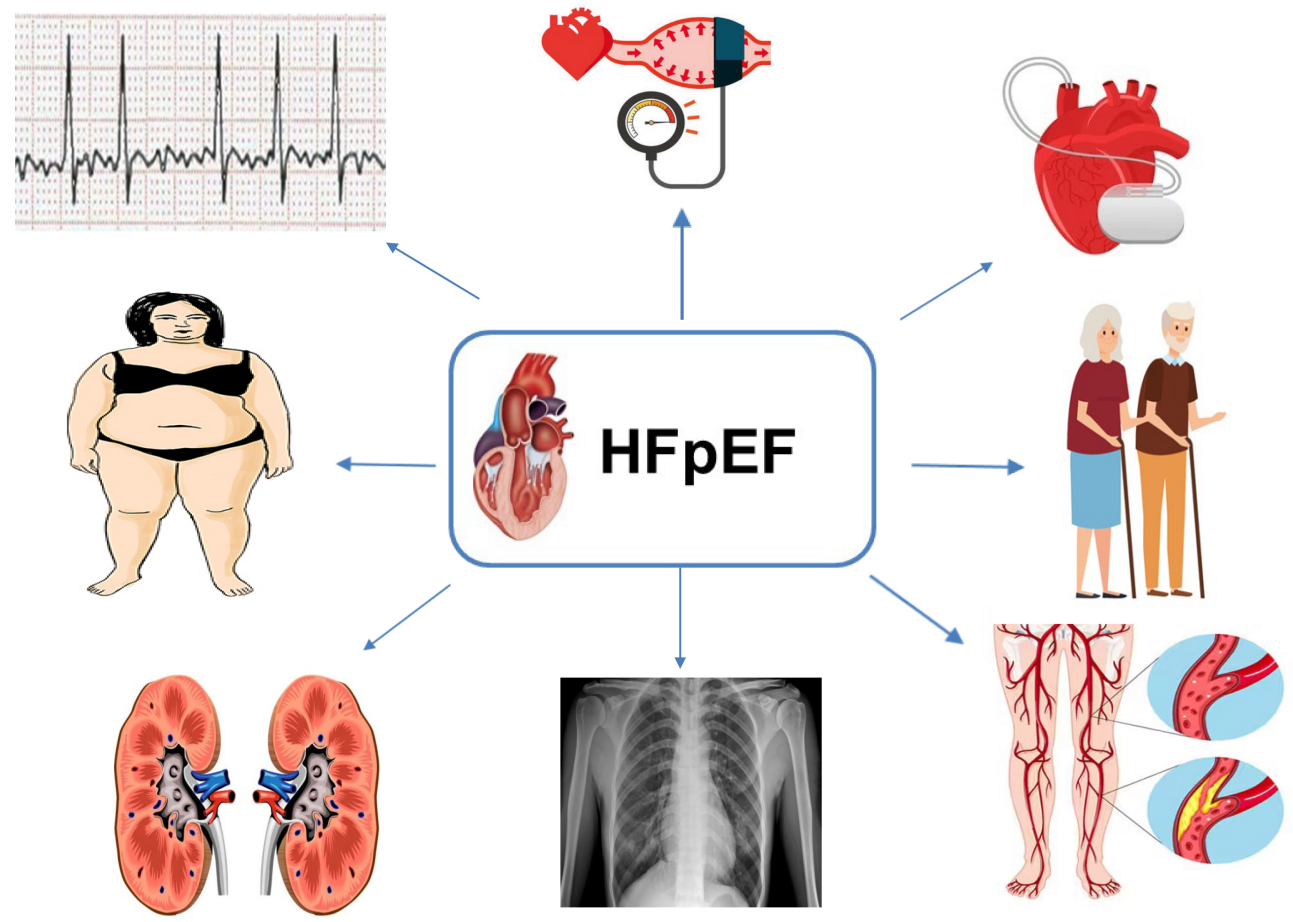
**Different HFpEF phenotypes and clinical scenarios may respond differently to treatment. **HFpEF, heart failure with preserved ejection fraction.

Five distinct groups were chosen by another analysis based on shared laboratory characteristics: Age, gender, and related disorders, which came from the SwedHF registry. Similarly to the previous analysis, Cluster 1 included younger patients with low comorbidities and NP values. Cluster 2 encompasses patients with a high prevalence of AF and diabetes associated with impaired renal function; Cluster 3 demonstrated a higher incidence of AF, older age, the prevalence of females, and those associated with a high NP level; Cluster 4 could be identified by the elevated prevalence of hypertension and diabetes with high body mass index (BMI); Cluster 5 identified older patients with cardio–renal disorders and more advanced New York Heart Association (NYHA) classes. The current classifications show a poorer outcome among groups with cardio–renal and hypertension–diabetic patterns [39]. Similarly, an analysis of the Alberta cohort recognized four phenotypes ranging from healthy subjects at risk of HFpEF without signs of HF up to congested, older patients with elevated comorbidity burdens [40]. The more recent Prospective Comparison of angiotensin receptor–neprilysin inhibitor (ARNI) with angiotensin receptor blocker (ARB) Global Outcomes in HFpEF (PARAGON-HF) included older individuals with a mean age of 72 years, a mean EF of 57%, a significant prevalence of diabetes (43%), and chronic kidney disease (47%), reflecting the real HFpEF patients [41]. Looking at more detailed morphological investigations, these patients had similar LA sizes but lower evidence of increased LV mass values and tricuspid regurgitation velocity compared to previous trials. Conversely, the E/e’ ratio in the PARAGON trial was more noticeable, emphasizing the variability in the cardiac phenotype associated with this disease [42]. The comparison of this research highlights the differences in patient characteristics, especially the observation that only half of the people enrolled in the most well-known trials met the HFpEF diagnostic criteria. Interestingly, 50% of patients exhibited increased LV mass, yet while advanced diastolic dysfunction was common, treatment with sacubitril/valsartan was the most beneficial for those with an EF below normal [43] (Fig. 1).

Current discrepancies demonstrated that different mechanisms may primarily be responsible for disease onset and progression depending on different baseline conditions, burden of risk factors, and cardiovascular adaptation to the underlying triggers [44]. A certain amount of overlap and negligence between phenomapping analyses may reflect different HFpEF phases and selection criteria. Comprehensive morphological analysis, combined with environmental lifestyle and CV risk factors, may result in a more homogeneous classification and improved risk stratification of HFpEF [45, 46].

Screening and treating underlying causes and comorbidities, both cardiovascular and non-cardiovascular, are advised for HFpEF patients.

When AF causes HF, the clinical course tends to be more favorable compared to other causes of HF (known as tachycardiomyopathy) [47]. Nevertheless, the presence of AF may attenuate the prognostic benefits associated with beta-blockers and impede the efficacy of ivabradine [48]. Certain HF treatments, such as angiotensin-converting enzyme inhibitors (ACE-I), may reduce the risk of developing AF.

To manage HFpEF, beta-blockers, long-acting nitrates, calcium channel blockers (CCBs), ivabradine, ranolazine, trimetazidine, nicorandil, and their associations should be contemplated for angina relief, notwithstanding the absence of demonstrable benefits on HF progression or coronary endpoints [49, 50, 51, 52, 53].

Hypertension has emerged as a key driver of HFpEF, posing a significant treatment challenge with uncertain optimal approaches.

ARBs, ACE-I, and CCBs cause more effective LVH regression than beta-blockers or diuretics. However, caution is warranted to prevent hypotension in HFpEF patients with LVH and limited preload reserves.

Further, obesity strongly correlates with HFpEF, revealing distinct pathophysiological pathways in obese patients [8]. Weight reduction and increased physical activity may enhance symptom management and exercise capacity, especially in suitable candidates [54].

Chronic kidney disease (CKD) and declining renal function are more prevalent in HFpEF patients compared to heart failure with mildly reduced ejection fraction (HFmrEF) and HFrEF; however, their impact on outcomes appears less severe in HFpEF relative to the other classifications [55].

Management of HF in chronic obstructive pulmonary disease (COPD) patients generally proceeds without major concerns. While beta-blockers may exacerbate pulmonary function in select cases, they are not contraindicated in COPD or asthma [56, 57].

## 4. ACE-I, ARBs, Beta-Blockers and Diuretics in HFpEF

Before the EMPagliflozin outcomE tRial in patients with chrOnic heaRt failure (EMPEROR-Preserved) and Dapagliflozin Evaluation to Improve the LIVEs of Patients with Preserved Ejection Fraction Heart Failure (DELIVER) studies, American and European guidelines lacked recommendations for the use of disease-modifying HFrEF therapies as clinical trials with ACE-I, ARB, mineralocorticoid receptor antagonists (MRA), while ARNIs failed to achieve their primary endpoints in HFpEF patients.

Notable trials include PEP-CHF (perindopril) [58], CHARM-Preserved (candesartan) [59], I-PRESERVE (irbesartan) [60], TOPCAT (spironolactone) [61], DIG-Preserved (digoxin) [62], and PARAGON-HF (sacubitril/valsartan) [63]. Candesartan and spironolactone showed reductions in hospitalizations for heart failure, with a trend toward reductions observed while using sacubitril/valsartan. However, since these trials were neutral for their primary endpoints, these findings are considered hypothesis-generating only.

In the SENIORS trial, although nebivolol significantly decreased the combined primary endpoints of all-cause mortality and CV hospital admission, it only included 15% of participants with an LVEF greater than 50% [64].

Despite no treatment showing conclusive evidence of reducing mortality and morbidity in HFpEF patients, most of them have underlying hypertension and/or coronary artery disease (CAD), and many have already been treated with ACE-I/ARB, beta-blockers, or MRAs.

In congested patients with HFpEF, diuretics are recommended to mitigate symptomatic distress and clinical manifestations. Loop diuretics are preferred, while thiazide diuretics may also prove efficacious in managing hypertension.

## 5. ARNIs Action Mechanisms in HFpEF

The action mechanisms of ARNIs in HFpEF involve a multifaceted approach targeting various pathological processes, including neurohormonal activation, inflammation, and fibrosis. ARNIs represent a relatively novel class of drugs that combine the actions of ARBs and neprilysin inhibitors. The primary components of ARNIs are sacubitril and valsartan, combined in a unique crystalline salt complex with high stability and good pharmacodynamics. Sacubitril inhibits neprilysin, an enzyme responsible for degrading NPs, which has beneficial effects on blood pressure regulation and sodium balance. Conversely, valsartan is an ARB that blocks the effects of angiotensin II, a vasoconstrictor and pro-fibrotic peptide. Their combination is crucial because increasing the concentration of circulating neprilysin and sacubitril causes a reflex renin-angiotensin-aldosterone (RAAS) activation that limits its benefit, while valsartan inhibits this reflex [65].

**Fig. 2.**
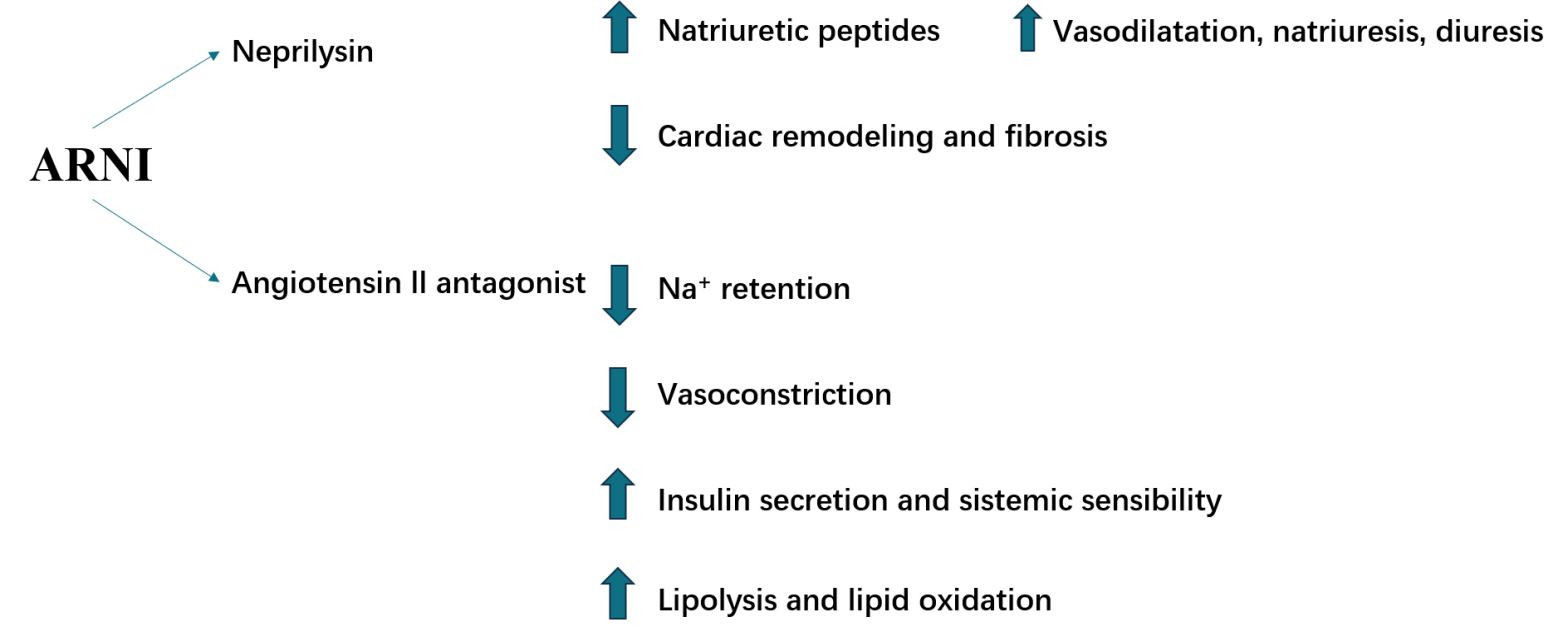
**ARNIs have potential beneficial molecular effects in HFpEF acting through different molecular mechanisms.** ARNI, angiotensin receptor–neprilysin inhibitor; HFpEF, heart failure with preserved ejection fraction.

The potential beneficial mechanisms of ARNIs in HFpEF are multifaceted:

• NP enhancement: By inhibiting neprilysin, ARNIs increase the levels of NPs, such as atrial natriuretic peptide (ANP) and B-type natriuretic peptide (BNP). These peptides promote vasodilation, natriuresis, and diuresis, which help reduce fluid overload and improve renal function.

• Angiotensin II blockade: Valsartan blocks the effects of angiotensin II, contributing to vasoconstriction, sodium retention, and myocardial remodeling.

• Improvement in cardiac remodeling: In HF, cardiac remodeling is often caused by microvascular inflammation and is often mediated by a reduction of intracellular cyclic guanosine monophosphate (cGMP). Increasing cGMP neprilysin counteracts this action, reducing left ventricular remodeling, myocardial hypertrophy, and fibrosis, thus reducing cardiomyocyte stiffness and ultimately improving cardiac function and structure.

• Beneficial metabolic effects: ARNIs act in both lipid and carbohydrate metabolism, increasing lipolysis and lipid oxidation, insulin secretion, and insulin sensitivity through many pathways. This is crucial because metabolic dysregulation is an important risk factor for HFpEF, especially affecting cardiac diastolic function [66] (Fig. 2).

## 6. Clinical Trials on ARNIs in HFpEF

HFpEF management is still a topic of debate because of the lack of specific therapies, even if many drugs designed for HFrEF have also been investigated for HFpEF. According to both the 2021 ESC guidelines and the 2022 American College of Cardiology (ACC)/American Heart Association (AHA) guidelines on HF, ARNIs may be considered in HFpEF (Class IIB) [11, 67]. Unfortunately, the class of recommendation is still low because, to date, clinical trials have failed to show the benefit of these drugs in hard endpoints such as cardiovascular deaths or hospitalizations.

In 2012, the Prospective Comparison of ARNI with ARB on the Management of Heart Failure with Preserved Ejection Fraction (PARAMOUNT) was the first trial that clearly demonstrated the potential role of ARNIs in HFpEF. PARAMOUNT is a phase 2 randomized double-blind trial comparing sacubitril/valsartan to valsartan alone in 301 patients with HFpEF (EF ≥45%) for a 36-week follow-up. It demonstrated a reduction in NT-proBNP levels in the sacubitril/valsartan group after 12 weeks, with an improvement in NYHA class and a reverse LA remodeling at 36 weeks [68]. Despite these results, subsequent trials have been controversial. In 2019, three important trials were published: the PARAGON-HF, the Comparison of Sacubitril/Valsartan Versus Enalapril on Effect on NT-proBNP in Patients Stabilized from an Acute Heart Failure Episode (PIONEER-HF), and the Prospective Comparison of ARNI vs. Comorbidity-Associated Conventional Therapy on Quality of Life and Exercise Capacity (PARALLAX) trials [63, 69, 70].

Using the same study groups as the PARAMOUNT trial but with 4822 patients, who were observed for a median follow-up of 35 months, the PARAGON-HF trial did not show a benefit in deaths or hospitalizations among sacubitril/valsartan patients, despite an improvement in the NYHA class and the quality of life, measured using the Kansas City Cardiomyopathy Questionnaire (KCCQ). Nevertheless, a subgroup analysis suggested potential ARNI survival benefits in women and patients with lower EF (45–57%). Furthermore, the benefit of ARNIs was larger in patients recently hospitalized for HF [63].

The PARALLAX trial with 2572 patients, EF >40%, and a median follow-up of 24 weeks showed a reduction in NT-proBNP in patients treated with sacubitril/valsartan compared to patients treated with an ACE-I or ARB. However, there was no significant improvement in the NYHA class, KCCQ, or the 6-minute walk distance [69]. Compared to the PARAMOUNT trial, these findings suggest that NT-proBNP reduction is probably an early effect of ARNI treatment, while other benefits are shown later. Furthermore, the ARNI effects on NT-proBNP are not limited to a chronic setting but are also important in acute HF, as shown by the PIONEER-HF trial in HFrEF [70] and by the Prospective comparison of ARNI with ARB Given following the stabiLization In DEcompensated HFpEF (PARAGLIDE) trial. However, also in the PARAGLIDE trial, sacubitril/valsartan did not reduce mortality, even if the hierarchical outcome showed a positive but not significant trend (unmatched win ratio: 1.19; 95% CI: 0.93–1.52; *p* = 0.16) [71] (Table 2, Ref. [63, 68, 69, 71]).

**Table 2.** **Main randomized clinical trials on ARNIs in patients with HFpEF with improved soft endpoints but no clear effect on CV deaths**.

Study	Year	Drugs	Patients	EF	Primary endpoint	*p*-value	Follow-up
PARAMOUNT	2012	Sacubitril/valsartan vs. valsartan	149	≥45%	Change from baseline in NT-proBNP level at week 12 [68]	*p* = 0.01	36 weeks
PARAGON-HF	2019	Sacubitril/valsartan vs. valsartan	4822	≥45%	Composite of total hospitalizations for HF and death from CV causes [63]	*p* = 0.06	35 months
PARALLAX	2021	Sacubitril/valsartan vs. enalapril, valsartan, or placebo	4632	>40%	Change from baseline in plasma NT-proBNP level at week 12.	*p* < 0.001	24 weeks
					Change from baseline in the 6-minute walk distance at week 24 [69]	*p* = 0.42	
PARAGLIDE	2023	Sacubitril/valsartan vs. valsartan	466	>40%	Time averaged reduction in NT-proBNP after an acute HF episode [71]	*p* = 0049	8 weeks

ARNI, angiotensin receptor–neprilysin inhibitor; CV, cardiovascular; EF, ejection fraction; HF, heart failure; PARAMOUNT, Prospective Comparison of ARNI with ARB on Management of Heart Failure with Preserved Ejection Fraction; PARAGON-HF, Prospective Comparison of ARNI with ARB Global Outcomes in HFpEF; PARALLAX, Prospective Comparison of ARNI vs. Comorbidity-Associated Conventional Therapy on Quality of Life and Exercise Capacity; PARAGLIDE, prospective comparison of ARNI with ARB Given following stabiLization In DEcompensated HFpEF; HFpEF, heart failure with preserved ejection fraction; NT-proBNP, N-terminal pro-B-type natriuretic peptide; ARB, angiotensin receptor blocker.

In conclusion, the role of ARNIs in HFpEF is still debated; however, it seems to lower HF hospitalizations in HFpEF patients [72]. Furthermore, a recent pooled analysis of the PARAGON and PARAGLIDE trials showed a significant reduction in deaths or hospitalizations in patients treated with sacubitril/valsartan compared to valsartan alone, with a larger benefit in patients with LVEF ≤60% [73].

## 7. MRAs Action Mechanisms in HFpEF

**Fig. 3.**
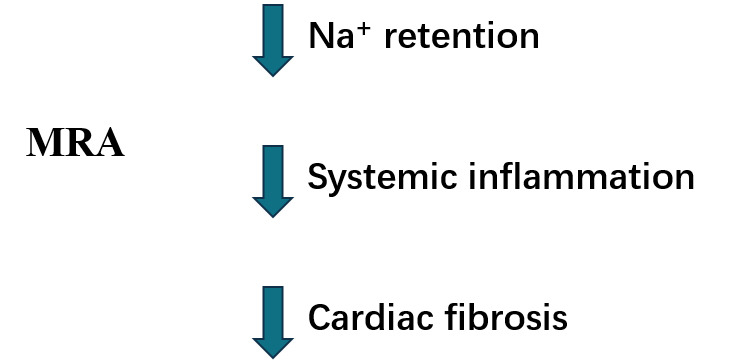
**MRA potential beneficial mechanisms in HFpEF.** MRA, mineralocorticoid antagonists; HFpEF, heart failure with preserved ejection fraction.

MRAs, such as spironolactone and eplerenone, antagonize mineralocorticoid receptors, reducing aldosterone effects that can harm HFpEF, promoting sodium and water retention, and exacerbating cardiac fibrosis and inflammation. The role of MRAs in HFpEF should probably be discussed alongside HF pathophysiology. Indeed, MRAs are probably more effective in patients with hypertension as an etiology of HF [74]. Their potential beneficial mechanisms in HFpEF include:

• Sodium and water balance: MRAs counteract sodium and water retention caused by aldosterone, reducing edema and congestion.

• Reduction in myocardial fibrosis: Aldosterone is implicated in cardiac fibrosis; thus, MRAs may have a role in reducing myocardial remodeling.

• Anti-inflammatory effects: MRAs may have anti-inflammatory properties, which could help ameliorate the systemic inflammation often seen in HFpEF patients [75, 76] (Fig. 3).

## 8. Clinical Trials on MRAs in HFpEF

As for ARNIs, MRAs could also be used in HFpEF according to the latest ESC guidelines and ACC/AHA guidelines on HF (Class IIB) [11, 67]. However, their effects on CV deaths and hospitalizations remain uncertain and under investigation.

In 2013, the Aldosterone Receptor Blockade in Diastolic Heart Failure (ALDO-HF) trial showed that E/e’ and reduced left ventricular mass in the treatment group (n = 213) compared to the placebo (n = 209), even if it did not benefit the maximal exercise capacity or the patient’s symptoms and quality of life [77]. In 2014, the Treatment of Preserved Cardiac Function Heart Failure with an Aldosterone Antagonist trial showed a decrease in hospitalizations but not in mortality in patients with HFpEF [61, 78], even though a subsequent sub-analysis seems to illustrate a survival benefit in the American cohort of the trial compared to the Russian cohort; differences in drug compliance have been hypothesized to clarify these results [79]. Furthermore, in 2016, the Spironolactone in Myocardial Dysfunction with Reduced Exercise Capacity (STRUCTURE) trial showed an improvement in exercise capacity using the cardiopulmonary test in patients with HFpEF and exertional dyspnea [80].

Currently, the only large study on MRAs with positive results on the survival rate is the Canrenone Effects on Cardiovascular Mortality in Patients with Congestive Heart Failure (COFFEE-IT) trial, a retrospective study that showed the survival benefit of canrenone on older people (68–83 years old) after 10 years of treatment [81]. Despite this, canrenone remains poorly used among MRAs.

Results from two large trials investigating the impact of spironolactone on cardiovascular deaths and hospitalizations should be published in the near future from the Spironolactone Initiation Registry Randomized Interventional Trial in Heart Failure with Preserved Ejection Fraction (SPIRRIT-HF) [82] and the Spironolactone In The Treatment of Heart Failure (SPIRIT-HF) trials [83]. Furthermore, compared to traditional MRAs, non-steroidal MRAs are emerging as drugs with higher selectivity for mineralocorticoid receptors and without sex–steroid-related side effects [84]. Among them, finerenone is the most investigated drug, and subgroup analyses of the FIDELIO-DKD [85] and FIGARO-DKD on patients with HF (symptomatic HFrEF was an exclusion criterion in both trials) suggest a potential role of this drug in HFpEF [86]. Indeed, a reduction in the composite outcome of cardiovascular death, non-fatal myocardial infarction, stroke, or hospitalization for heart failure was observed in a mean follow-up of 2.6 years. As a result, the Finerenone in Heart Failure Patients (FINEARTS-HF) trial is currently ongoing, investigating finerenone effects on morbidity in more than 6000 patients with HF and EF ≥40% [87] (Table 3, Ref. [77, 79, 80, 82, 83, 87]).

**Table 3.** **Main randomized clinical trials on MRAs in patients with HFpEF with improved secondary endpoints but no clear effect on CV deaths**.

Study	Year	Drugs	Patients	EF	Primary endpoint	*p*-value	Follow-up
ALDO-HF	2013	Spironolactone vs. placebo	422	≥50%	Change in diastolic function (E/e’).	*p* < 0.001	12 months
					Change in peak oxygen uptake on cardiopulmonary exercise testing [77]	*p* = 0.81	
TOPCAT	2014	Spironolactone vs. placebo	3445	≥45%	Composite of death from CV causes, aborted cardiac arrest, or hospitalization for HF [79]	*p* = 0.014	3.3 years
STRUCTURE	2016	Spironolactone vs. placebo	150	>50%	Improvement in peak oxygen uptake.	*p* <0.001	6 months
					Improvement exertional E/e’ ratio [80]	*p* <0.001	
SPIRRIT-HF	On going	Spironolactone vs. no spironolactone	2000	≥40%	Incidence rate for total HF hospitalizations or CV death [82]		5 years
SPIRIT-HF	On going	Spironolactone vs. placebo	1300	≥40%	Cumulative number of primary composite events of CV death and total HF hospitalizations [83]		48 months
FINEARTS-HF	On going	Fineronone vs. placebo	6016	≥40%	Number of CV deaths and HF events [87]		42 months

CV, cardiovascular; EF, ejection fraction; HF, heart failure; MRAs, mineralocorticoids antagonists; HFpEF, heart failure with preserved ejection fraction; ALDO-HF, Aldosterone Receptor Blockade in Diastolic Heart Failure; TOPCAT, Treatment of Preserved Cardiac Function Heart Failure with an Aldosterone Antagonist; STRUCTURE, Spironolactone in Myocardial Dysfunction with Reduced Exercise Capacity; SPIRRIT-HF, Spironolactone Initiation Registry Randomized Interventional Trial in Heart Failure with Preserved Ejection Fraction; SPIRIT-HF, Spironolactone In The Treatment of Heart Failure; FINEARTS-HF, Finerenone in Heart Failure Patients; E/e’, spironolactone improved diastolic function.

## 9. Sodium–Glucose Cotransporter-2 Inhibitor (SGLT2i) in HFpEF: Potential Mechanism of Action and Results from Trials

Recently, the ESC guidelines recommended SGLT2i therapy for treating HFpEF [11], despite the exact mechanisms yet being fully understood. However, several potential mechanisms of action have been proposed based on clinical and pre-clinical evidence [85]. SGLT2i plays the potential role of anti-HFpEF through the direct or indirect synergy of multiple targets and pathways [88].

SGLT2i inhibits the absorption of sodium and glucose in the proximal renal tubule, leading to natriuresis, glucosuria, and elevated urine output. Thus, it has been associated with a reduction in blood pressure. Lowering blood pressure can alleviate cardiac workload and improve overall cardiovascular function.

The vascular and metabolic effects of SGLT2i have always been held responsible for cardiovascular benefits [89]. In HFpEF studies, researchers propose potent reno-protective effects of SGLT2i, with their impact on intraglomerular pressure prevailing over other mechanisms. SGLT2i, by restoring afferent arteriole tone, synergizes with renin-angiotensin system inhibitors, reducing intraglomerular pressure and preventing renal complications. Notably, despite a decline in the estimated glomerular filtration rate (eGFR), SGLT2 inhibition influences primary endpoints [90].

In a *post-hoc* analysis of the EMPEROR-Preserved Trial, empagliflozin was linked to a slight increase in the risk of volume depletion for patients concurrently using diuretics [91]. However, it was also associated with a decreased probability of initiating or escalating diuretic doses and an increased likelihood of reducing or permanently discontinuing diuretics [91]. Natriuresis derived from empagliflozin is not associated with neurohormonal activation, potassium loss, or impaired renal function (favorable diuretic profile) [92].

SGLT2i provides metabolic advantages, promotes weight loss, and enhances insulin sensitivity for improved cardiovascular health. It also exhibits intriguing effects, which include inducing a transcriptional paradigm, nutrient deprivation, hypoxia, increasing ketosis, erythropoietin, and autophagic flux [93]. Consequently, inflammasome activation is reduced, mitigating cardiomyocyte dysfunction and coronary microvascular injury. Moreover, alterations in iron homeostasis contribute to enhanced cardiac energetics and function. Additionally, SGLT2i reduces epicardial adipose tissue and modifies adipokine signaling, potentially contributing to the observed reductions in inflammation and oxidative stress associated with their use [93].

Dapagliflozin has a significant impact on energy metabolism related to fatty acid intake and mitochondrial dysfunction in HFpEF; it is achieved by elevating β-hydroxybutyric acid (β-OHB) levels, activating citrate synthase, reducing acetyl coenzyme A (acetyl-CoA) pools, regulating adenosine 5’-triphosphate production, and enhancing the expression of mitochondrial oxidative phosphorylation system complexes I–V. SGLT2i is beneficial in preventing and treating cardiac remodeling and dysfunction in HFpEF models by mitigating cardiometabolic dysregulation [94].

Endothelial dysfunction is a pivotal mechanism in HFpEF, diabetes mellitus (DM), and frailty. Treatment with the SGLT2i empagliflozin altered certain microRNAs, counteracting the changes observed in HFpEF patients. This suggests a potential restoration of endothelial function through empagliflozin treatment [95].

Chronic activation of the SGLT2i pathway may contribute to maladaptive cardiac remodeling. Inhibiting SGLT2 receptors could potentially mitigate adverse remodeling processes, improving cardiac structure and function [96].

SGLT2i can affect the extracellular matrix, contributing to collagen turnover and mitigating fibrosis—a significant feature in HFpEF. Notably, these inhibitors have demonstrated a capacity to reduce myofilament stiffness and remodel the extracellular matrix in the heart. This action improves diastolic function, offering potential benefits in the context of HFpEF [93].

Canagliflozin (CANA) treatment reduces myocardial hypertrophy and fibrosis and improves left ventricular diastolic function and remodeling. These positive effects are attributed to CANA’s ability to upregulate apelin, activate angiotensin-converting enzyme 2 (ACE2), and increase ACE2/Ang (1–7)/mast cell receptor (MASR) axis levels [97]. Canagliflozin treatment was found to counteract ferroptosis, a recently identified mechanism of iron-dependent non-apoptotic cell death in HF. In a rat model of HF induced by a high-salt diet, the study observed iron overloading and lipid peroxidation, both of which had been alleviated by administering canagliflozin [98]. SGLT2i mitigates the risk of hospitalization for HF in individuals with HFpEF. However, the specific hemodynamic mechanisms responsible for these benefits are poorly understood. In the CAMEO-DAPA trial, dapagliflozin treatment in HFpEF patients was associated with reduced resting and exercise pulmonary capillary wedge pressure (PCWP) and positive effects on plasma volume and body weight [99].

Emerging evidence also indicates that these drugs impact cardiomyocyte ionic homeostasis. Empagliflozin was observed to diminish the activity of the cardiac ${\mathrm{Na}}^{+}$/$H^{+}$ exchanger, potentially enhancing cardiac function. Subsequently, it was discovered that dapagliflozin and canagliflozin also inhibited ${\mathrm{Na}}^{+}$/$H^{+}$ exchanger activity, leading to a decrease in cytosolic ${\mathrm{Na}}^{+}$. Moreover, empagliflozin reduced the activity of ${\mathrm{Ca}}^{2+}$/calmodulin-dependent kinase II (CaMKII) and CaMKII-dependent sarcoplasmic reticulum ${\mathrm{Ca}}^{2+}$ leakage [100].

The effects of empagliflozin on HFpEF are primarily mediated by inhibiting ${\mathrm{Na}}^{+}$/$H^{+}$ exchanger 1 (NHE1), influencing cardiomyocyte oxidative stress modulation, cardiomyocyte stiffness, myocardial extracellular matrix remodeling, heart concentric hypertrophy, and systemic inflammation [101]. Empagliflozin enhances the nitric oxide (NO)—soluble guanylate cyclase (sGC)—cGMP cascade and protein kinases GIα (PKGIα) activity by reducing PKGIα oxidation in HFpEF [102]. Additionally, dapagliflozin inhibits the inflammatory response and activates the NO–cGMP–protein Kinases G (PKG) pathway in animal models [103].

**Fig. 4.**
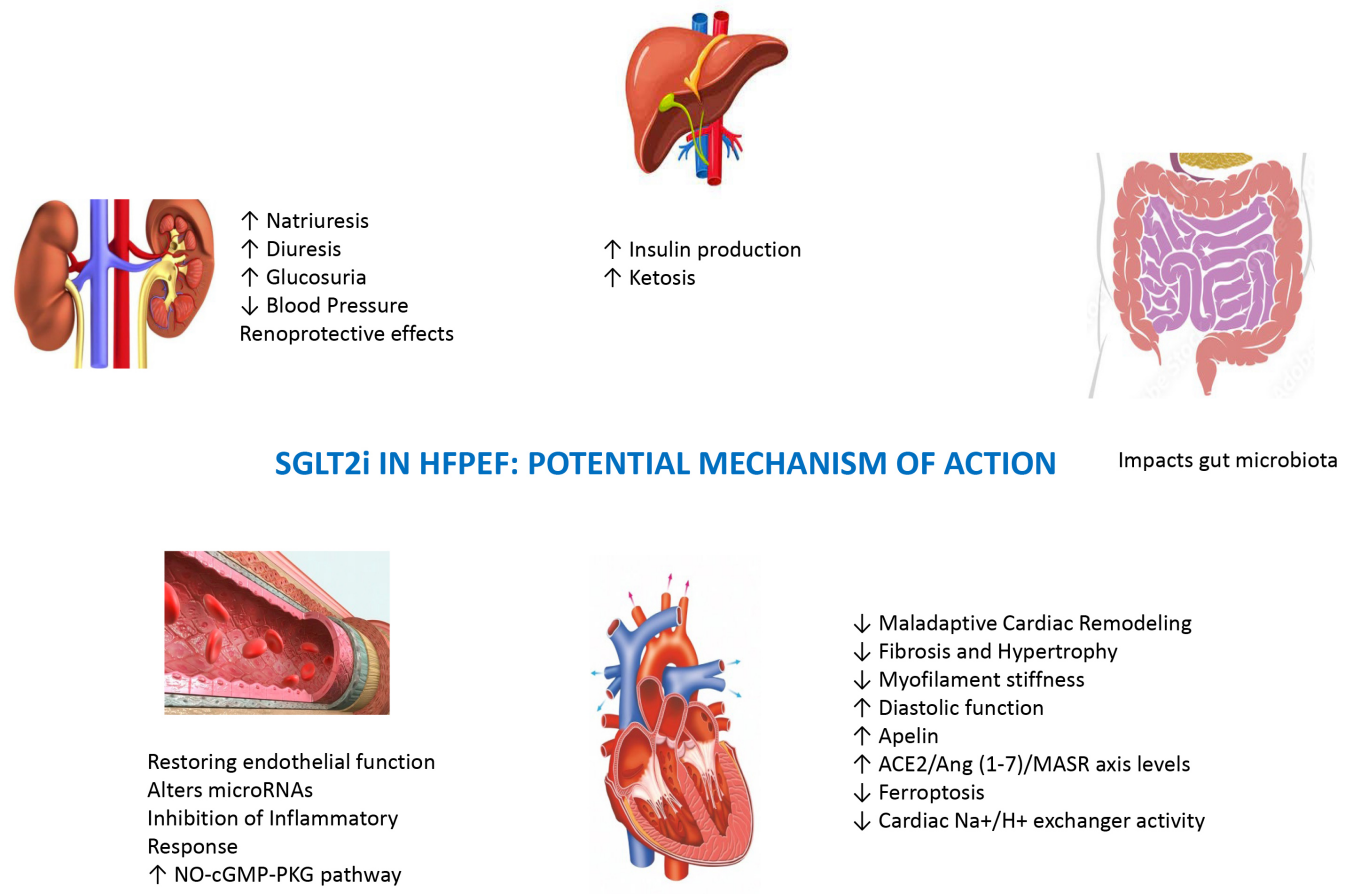
**Potential SGLT2i effects beyond the cardiovascular system on renal, liver, and intestinal districts. **SGLT2i, sodium–glucose cotransporter-2 inhibitors; HFpEF, heart failure with preserved ejection fraction; ACE2, angiotensin-converting enzyme 2; NO, nitric oxide; cGMP, cyclic guanosine monophosphate; MASR, mast cell receptor; PKG, protein Kinases G.

Prior evidence showed that metabolites produced by gut microbiota play a crucial role in heart failure development. SGLT2i have been discovered to impact the gut microbiota in rodent studies. The EMPAGUM trial seeks to validate these human changes and explores the role of gut microbiota and their metabolites in the HFpEF process [104]. It is important to note that ongoing research further elucidates the mechanisms of SGLT2i in HFpEF, and the field continues to evolve (Fig. 4).

The EMPEROR-Preserved study study was carried out on 5988 patients (median age: 72, 45% women, median LVEF: 54%); half of the patients had diabetes, and half had an eGFR below 60 mL/min/1.73 $m^{2}$. The primary endpoint (cardiovascular events and HF hospitalization) was met (hazard ratio (HR) 0.73; 95% confidence interval (CI), 0.6–0.88; *p* <0.001), marking the first instance where drug therapy achieved this goal in an HFpEF study.

The trial validated a 21% risk reduction in cardiovascular death or HF hospitalization from using empagliflozin on HF patients with an LVEF >40% [105]. Subgroup analysis has shown the highest benefit in LVEF <50% (HR 0.71; 95% CI, 0.57–0.88), a lower benefit in LVEF 50 to <60% (HR 0.80; 95% CI, 0.64–0.99), and no apparent benefit in LVEF ≥60% (HR 0.87; 95% CI, 0.69–1.10). Pooled analysis across EMPEROR-Reduced and EMPEROR-Preserved trials have indicated consistent HF outcomes with empagliflozin in patients with LVEF <25% to <65%, with diminished effects in those with LVEF ≥65% [106]. A potential drawback in the EMPEROR-Preserved trial was raised from the under-representation of females and a predominantly low NYHA class within the studied population. Additionally, there was no observed benefit of empagliflozin on overall mortality, as indicated by a hazard ratio of 1 (95% CI, 0.87–1.15).

In the Dapagliflozin Evaluation to Improve the LIVEs of Patients with Preserved Ejection Fraction Heart Failure trial of 6263 heart failure patients (mean age: 71, 44% female, mean LVEF: 54%), dapagliflozin led to an 18% risk reduction in the primary combined endpoint (HR 0.82; 95% CI, 0.73–0.92) and a non-significant 12% reduction in cardiovascular mortality (HR 0.88; 95% CI, 0.74–1.05) [107] over 2.3 years. Dapagliflozin lowered the risk of HF worsening or cardiovascular death by 18%, regardless of LVEF. Unlike prior trials, DELIVER included all LVEF ranges, but it is unclear if the benefit in patients with LVEF >60% specifically relates to a reduction in total CV events, HF events, or both.

DELIVER permitted randomization during or soon after hospitalization for HF in clinically stable patients without intravenous HF therapies. Starting dapagliflozin during, or soon after, HF hospitalization in patients with mildly reduced or preserved LVEF has appeared safe and effective; moreover, dapagliflozin has reduced the risk of worsening HF or cardiovascular death similarly in patients with and without a history of recent HF hospitalization [108]. In patients recently hospitalized with HF, initiating dapagliflozin has had trivial effects on blood pressure and has not worsened renal status [109].

In a meta-analysis [110] performed to investigate SGLT2i effects on HFpEF or HFmrEF, by pooling data from all clinical RCTs available and thus increasing power to testify, the authors have demonstrated that in patients with LVEF >40%, SGLT2i significantly reduces the composite risk of cardiovascular death and hospitalization for heart failure, although the risk of cardiovascular death and all-cause death did not reduce.

## 10. Rationale for the Use of Glucagon-Like Peptide 1 Receptor Agonists

Type 2 diabetes mellitus (T2DM) and obesity could be considered metabolic disorders characterized by high CV risk [111].

Recently, a multinational, cross-sectional study of cardiovascular disease prevalence and etiology in adults with T2DM across 13 countries (CAPTURE study) showed that, among 9823 T2DM patients, one-third have a CV disease [112]. Moreover, the overall mean BMI was 29.0 ${\mathrm{kg/m}}^{2}$, highlighting a pathophysiological link between T2DM and obesity [112]. Importantly, according to U.S. National Health Interview Survey data, overall mortality in T2DM patients has reduced from 11.3% during 1988–1994 to 5.9% during 2010–2015. However, despite a paradigm shift in T2DM treatment beyond the hypoglycemic effect, the overall prevalence of CV complication is 32.2%, consisting mainly of CAD [113, 114, 115].

Obesity and T2DM are very common comorbidities in HFpEF patients and are closely involved in their pathophysiology and prognosis [116, 117]. T2DM-related metabolic derangements such as hyperglycemia, lipotoxicity, and hyperinsulinemia, associated with coronary microvascular rarefaction and advanced glycation end-products deposition, favor the development of DM-related cardiomyopathy (DMC) with HFpEF phenotype and concentric LV remodeling with diastolic LV dysfunction. Importantly, this phenotype is more prevalent in obese patients with T2DM [118]. Recently, a new paradigm of HFpEF syndrome has been developed: Particularly, HFpEF comorbidities are believed to induce a chronic systemic inflammatory state. In obese subjects, macrophages infiltrate adipose tissue, releasing proinflammatory cytokines. This condition promotes coronary microvascular endothelial inflammation, leading to a reduction in NO bioavailability. Reductions in both cGMP and protein kinase G activity increase wall tension, myocardial stiffness, and interstitial fibrosis [119]. These pathophysiological aspects suggest why innovative drugs targeting endothelial dysfunction are crucial in treating T2DM and obese patients and could be useful in diabetic and obese HFpEF phenotypes.

## 11. Mechanisms of Action of Glucagon-Like Peptide 1 Receptor Agonists and Their Potential Role in HFpEF

Incretin hormones—glucose-dependent insulinotropic polypeptide (GIP) and glucagon-like peptide 1 (GLP1)—are released from gut endocrine cells and potentiate meal-stimulated insulin secretion [120]. Biologically active GLP1 refers to both GLP1 (7–36) amide and GLP1 (7–37), which act on a single identified GLP1 receptor (GLP1R) on pancreatic islet β-cells, δ-cells, and α-cells, respectively, to increase insulin and somatostatin secretion and decrease glucagon secretion.

Synthetic GLP1R agonists (GLP1RAs) mediate the same biological effects of endogenous GLP1, binding to the GLP1R and stimulating glucose-dependent insulin release from the pancreatic islets.

A single, canonical GLP1R, expressed at low levels in the human atria and ventricles (including in cardiomyocytes) and in blood vessels, mediates the major CV actions of GLP1RAs, reducing blood pressure and improving microvascular and coronary flow, counteracting atherosclerosis, and promoting plaque stability [121]. Specifically, GLP1RAs might reduce myocardial apoptosis and inflammation in the heart, inducing glucose metabolism [120]. In addition, GLP1 and GLP1RAs have multiple extra-pancreatic effects that might indirectly reduce CV morbidity, inducing weight loss and acting on postprandial lipemia and inflammation [122]. Enterocytes and hepatocytes do not express GLP1R, although studies on mouse liver showed that a subset of GLP1R+ endothelial and intrahepatic γδ T cells mediates a component of the anti-inflammatory effect of GLP1RAs [123]. As proof of this, patients with either T2DM, obesity, or non-alcoholic steatohepatitis (NASH) treated with semaglutide had shown a decrease in circulating triacylglycerol, low density lipoprotein cholesterol (LDLc), and non-high density lipoprotein (HDL) cholesterol [124]. Similarly, several GLP1RAs were shown to reduce postprandial plasma levels of triacylglycerol with little effect on fasting plasma LDLc, even in patients treated with statins [125]. The effect of a reduction in the fasting plasma levels of cholesterol and triacylglycerol could indirectly reflect the extent of weight loss achieved in these patients [119]. Weight loss is determined by reducing hunger by stimulating the GLP1Rs expressed in the central nervous system and slowing down gastric emptying [120].

**Fig. 5.**
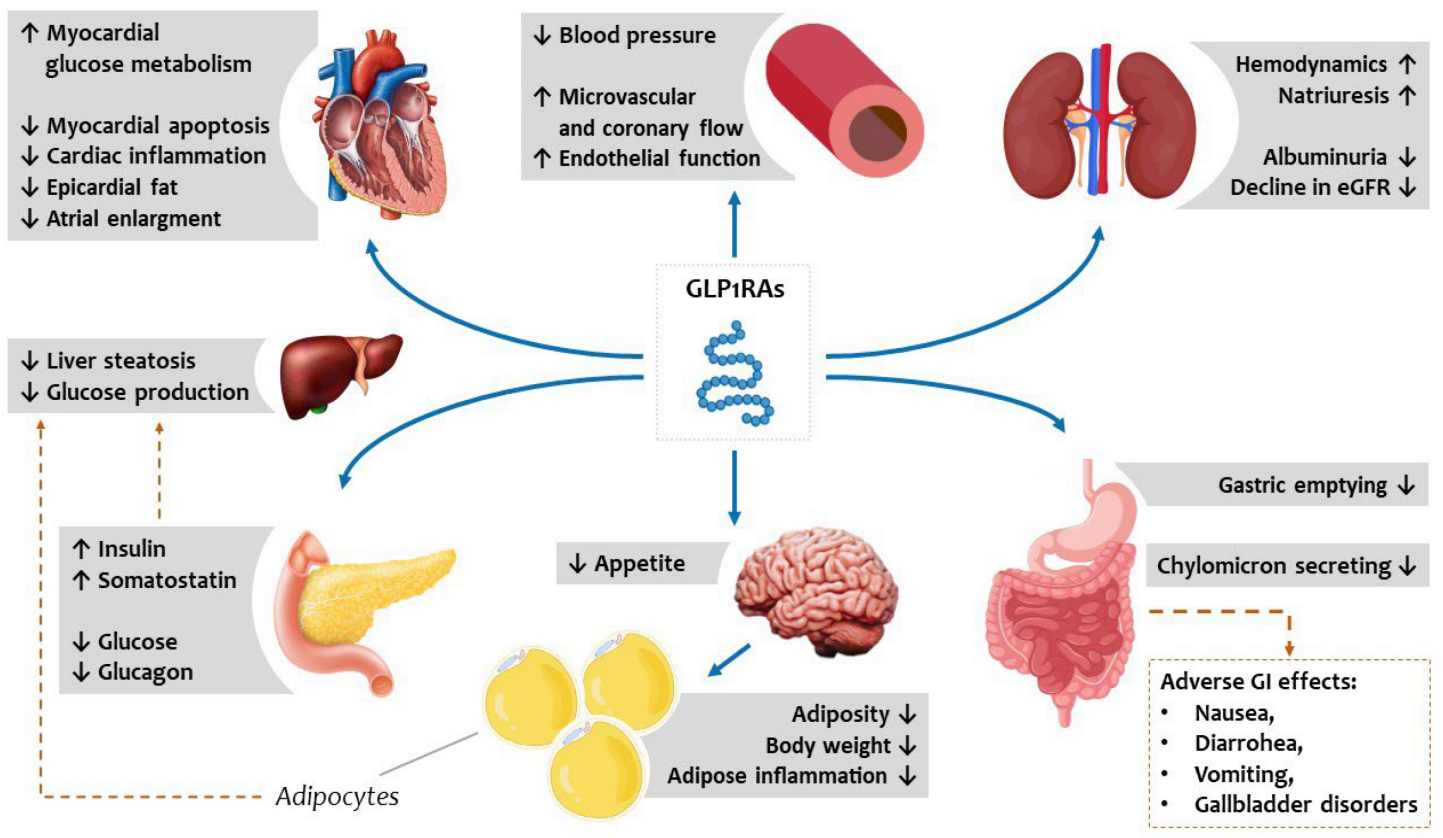
**Metabolic vascular and systemic effects of GLP1RAs potentially reduce the burden of cardiovascular risk and HFpEF deterioration.** GLP1RAs act directly on pancreatic beta and alpha cells, the gastrointestinal tract, and the central nervous system to improve glucometabolic homeostasis and indirectly improve circulating lipid profiles by reducing hepatic steatosis. GLP1RAs, synthetic glucagon-like peptide receptor agonists; HFpEF, heart failure with preserved ejection fraction; eGFR, estimated glomerular filtration rate; GI, gastrointestinal.

The activity of GLP-1 and GIP is limited by the dipeptidyl peptidase-4 (DPP-4) enzyme, which rapidly inactivates incretin hormones. This provides the rationale for developing DPP4 inhibitors, which limit the breakdown of endogenous GLP-1 by the DPP-4 enzyme. Concentrations of the active intact GLP-1 and GIP are therefore increased, leading to an increased and prolonged action of these hormones. The biological effects of GLP1RAs are summarized in Fig. 5.

GLP1RAs and DPP4 inhibitors were developed for T2DM treatment based on this evidence. However, these effects may be particularly relevant in the context of metabolic alterations and inflammation of HFpEF syndrome. As proof, GLP1RAs were shown to have protective effects in experimental HF models in several species, including mice, rats, pigs, and dogs, further suggesting a role in specific HFpEF phenotypes [119, 126]. The same molecule used in experimental animal models of T2DM cardiomyopathy improved diastolic dysfunction, as reflected by a decreased E/e’ ratio [127].

Recently, a meta-analysis of eight RCTs about CV events, mortality, and kidney outcomes with GLP1RAs in 60,080 T2DM patients showed a significant reduction (–14%) in major cardiovascular events (MACEs), all-cause mortality (–12%), and composite kidney outcome (–21%) with no increase in the risk of severe hypoglycemia, retinopathy, or pancreatic adverse events. Moreover, GLP1RAs significantly reduced hospitalizations for HF by 11% [128]. Effects on HF-related events were different depending on the patients treated. For example, a *post-hoc* analysis from Harmony Outcomes aiming to explore the effects of the GLP1RA albiglutide on HF outcomes in T2DM patients and cardiovascular disease, with and without HF history, showed that albiglutide had no effect in reducing HF-related events among those with a history of HF [129]. Similarly, in a *post-hoc* analysis from the REWIND trial, which included >5.4 years of follow-up, dulaglutide was not associated with a reduction in HF events in patients with T2DM regardless of baseline HF status [130].

Since the effects of GLP1RAs are mainly related to anti-inflammatory and lipolytic effects, their administration is associated with weight loss regardless of diabetic status [131]. Obesity and, more specifically, increased epicardial fat in HFpEF are associated with more symptoms and worse prognoses [132, 133, 134]. This has led to weight loss as a specific target for HFpEF treatment and the realted role of GLP1RAs in this context. However, the effect of weight loss in HF patients is still partially unsettled. Weight loss is a poor prognostic factor in patients with HFrEF [135, 136]. Conversely, evidence suggested that weight loss has beneficial effects in obese patients with HFpEF: Kitzman *et al*. [137] showed that among clinically stable obese older HFpEF patients, aerobic exercise training or caloric restriction increased peak oxygen consumption, and drug effects were additive [138]. Bariatric surgery was also shown to improve symptoms, as well as reduce HF rehospitalizations and reverse left ventricular remodeling in obese HFpEF patients [137, 139].

However, the same beneficial effects of weight loss have not been replicated in non-obese patients with HFpEF: the FLAGSHIP cohort study aimed to examine the association between weight loss and HFpEF prognosis in 573 hospitalized obese and non-obese patients [140]. In particular, in non-obese patients, weight loss was associated with higher all-cause mortality and rehospitalization rates than ones without weight loss. Moreover, 6 months after hospital discharge, a high proportion of non-obese patients with weight loss showed functional limitations and anorexia, suggesting an impairment of their physical function and poor nutritional status. Conversely, weight loss was not associated with adverse events in obese patients with HFpEF [141].

Based on these results, much evidence suggests that GLP1RAs, promoting weight loss by reducing the generation of reactive oxygen species and systemic inflammation, may be highly effective in the obese phenotype of HFpEF. Among GLP1RAs, semaglutide was shown to be more effective in weight loss compared with other agents: In the SUSTAIN trials, a slightly greater weight loss was observed with subcutaneous once-weekly semaglutide administration, compared with exenatide, dulaglutide, or liraglutide [142]. Similarly, a secondary analysis of PIONEER 4 showed a greater weight loss with once-daily oral semaglutide administration than with subcutaneous liraglutide [142, 143, 144, 145].

The STEP HFpEF trial has recently shown a significant improvement in symptoms, quality of life, and exercise tolerance, assessed by the 6-minute walking test (6MWT), along with weight loss, in 529 non-diabetic obese patients with HFpEF randomized to subcutaneous semaglutide (2.4 mg weekly) or placebo [140]. This trial also showed a significant reduction in C-reactive protein (CRP) in the semaglutide arm compared with the placebo. Importantly, a prespecified trial sub-analysis showed that semaglutide improved symptoms, physical limitations, and exercise function and reduced inflammation and body weight across obesity categories. Furthermore, the magnitude of benefit was directly related to the extent of weight loss (adjusting for age, sex, body weight at baseline, and other confounding variables) [146]. Semaglutide also greatly improved HF-related symptoms, physical limitations, exercise function, and NT-proBNP regardless of baseline health status [147]. In the STEP-HFpEF, semaglutide was also shown to reduce NPs. This evidence suggests a direct hemodynamic mechanism for semaglutide, regardless of caloric and metabolic improvement [148].

The STEP HFpEF DM trial (NCT04916470), which recently completed enrolment, will test the safety and benefit of semaglutide in obesity-related HFpEF patients with T2DM and explore the interaction with SGLT2i, as their use was rare in STEP-HFpEF but not in STEP HFpEF DM (32%) [140] (Table 4, Ref. [146, 147, 148, 149, 150]).

**Table 4.** **Characteristics of most recent clinical trials on semaglutide**.

Trial name, Author, Year	PIONEER 6, Husain *et al*. [149], 2019	STEP-HFpEF, Kosiborod *et al*. [146, 147, 148], 2023	SELECT, Lincoff *et al*. [150], 2023
Trial design	Event-driven, randomized, double-blind, placebo-controlled trial	Multinational (96 centers among 13 countries), double-blind, randomized, placebo-controlled clinical trial	Randomized, international multicenter, double-blind, placebo-controlled clinical trial
Study population	Patients with T2DM and:	Symptomatic patients with HFpEF (EF ≥45%), obesity (BMI ≥30 ${\mathrm{kg/m}}^{2}$) and without T2DM	Patients with overweight or obesity (BMI ≥ 27 ${\mathrm{kg/m}}^{2}$), established CVD (previous MI or stroke, or PAD) and without T2DM
	∙ ≥50 years old and CV disease or CKD, or		
	∙ ≥60 years old and CV risk factors only		
Intervention and control	Oral semaglutide (target dose, 14 mg) or placebo once-daily	Subcutaneous semaglutide (2.4 mg) or placebo once weekly	Subcutaneous semaglutide (2.4 mg) or placebo once weekly
Primary endpoint	∙ Time to the first occurrence of a MACE, a composite of death from CV causes, nonfatal myocardial infarction, or nonfatal stroke	∙ Change in KCCQ -CSS from baseline (week 0) to end of treatment (week 52)	∙ Composite of death from cardiovascular causes, nonfatal myocardial infarction, or nonfatal stroke, assessed in a time-to-first-event analysis
		∙ Change in body weight (%) from baseline (week 0) to end of treatment (week 52)	
Main secondary endpoints	Time to the first occurrence of the following: ∙ an expanded composite outcome consisting of the primary outcome plus unstable angina resulting in hospitalization or HF resulting in hospitalization ∙ a composite of death from any cause, nonfatal myocardial infarction, or nonfatal stroke ∙ the individual components of these composite outcomes	∙ Change in 6-MWD (meters) from baseline (week 0) to end of treatment (week 52) ∙ Hierarchical composite of time to all-cause death from baseline (week 0) to end of study (week 57) ∙ Hierarchical composite of number of HF events requiring hospitalization or urgent HF visit from baseline (week 0) to end of study (week 57) ∙ Hierarchical composite of time to first HF event requiring hospitalization or urgent HF visit from baseline (week 0) to end of study (week 57) ∙ Change in CRP (%) from baseline (week -2) to end of treatment (week 52)	Time-to-first-event analyses and tested in hierarchical order: ∙ Death from cardiovascular causes; ∙ A composite HF end point (death from cardiovascular causes or hospitalization or an urgent medical visit for HF); ∙ Death from any cause. Additional endpoints: ∙ Change in systolic blood pressure ∙ Change in body weight
Number of patients enrolled	3183; semaglutide (n = 1591) or placebo (n = 1592)	529; semaglutide (n = 263) or placebo (n = 266)	17,604; semaglutide (n = 8803) or placebo (n = 8801)
Median duration of follow up	64 weeks	52 weeks	160 weeks
Main results	Primary endpoint (semaglutide vs. placebo): ∙ MACEs: 3.8% vs. 4.8% (HR, 0.79; 95% CI, 0.57 to 1.11 *p* < 0.001 for noninferiority) Secondary endpoints (semaglutide vs. placebo): ∙ Death from CV causes: 0.9% vs. 1.9% (HR, 0.49; 95% CI, 0.27 to 0.92) ∙ Nonfatal myocardial infarction: 2.3% vs. 1.9% (HR, 1.18; 95% CI, 0.73 to 1.90) ∙ Nonfatal stroke: 0.8% vs. 1.0% (HR, 0.74; 95% CI, 0.35 to 1.57) ∙ First events of HF resulting in hospitalization: 1.3% vs. 1.5% (HR, 0.86; 95% CI, 0.48 to 1.55) ∙ Death from any cause: 1.4% vs. 2.8% (HR, 0.51; 95% CI, 0.31 to 0.84)	Co-primary endpoint (semaglutide vs. placebo): ∙ Change in KCCQ-CSS: 16.6 vs. 8.7 (*p* < 0.001) ∙ Percentage change in body weight: –13.3 vs. –2.6 (*p* < 0.001) Secondary endpoints (semaglutide vs. placebo): ∙ Change in 6-MWD from baseline to week 52: 21.5 vs. 1.2 m (*p* < 0.001) ∙ Percentage reduction from baseline to week 52 in NT-proBNP: –20.9 vs. –5.3 (*p* < 0.05) ∙ Hospitalization or urgent visit for HF: 1 vs. 12 events (*p* < 0.05) ∙ Reduction in CRP levels at week 52: 43.5 vs. 7.3 (*p* < 0.001) ∙ Percentage reduction in NT-proBNP level at week 52: –20.9 vs. –5.3 ∙ Adverse events were similar	Primary endpoint (semaglutide vs. placebo):
			∙ Composite of CV death, nonfatal MI, and nonfatal stroke, for semaglutide vs. placebo: 6.5% vs. 8.0% (HR 0.80, 95% CI 0.72–0.90, *p* < 0.001)
			Secondary endpoints (semaglutide vs. placebo):
			∙ CV death: 2.5% vs. 3.0% (HR 0.85, 95% CI 0.71–1.01, *p* = 0.07)
			∙ HF composite end point: 3.4% vs. 4.1% (HR 0.82, 95% CI 0.71–0.96)
			∙ All-cause death: 4.3% vs. 5.2% (HR 0.81, 95% CI 0.71–0.93)
			∙ Nonfatal MI: 2.7% vs. 3.7% (HR 0.72, 95% CI 0.61–0.85)
			∙ Hospitalization or urgent medical visit for HF: 1.1% vs. 1.4% (HR 0.79, 95% CI 0.60–1.03)
			Additional endpoints:
			∙ Change in systolic blood pressure: –3.8 vs. –0.5 mm Hg
			∙ Mean change in body weight at 104 weeks: –9.4% vs. –0.9%

BMI, body mass index; CI, confidence interval; CRP, C-reactive protein; CVD, cardiovascular disease; CKD, chronic kidney disease; HR, hazard ratio; KCCQ-CSS, Kansas City Cardiomyopathy Questionnaire-Clinical Summary Score; MACE, major cardiovascular event; MI, myocardial; HFpEF, heart failure with preserved ejection fraction; T2DM, type 2 diabetes mellitus; CV, cardiovascular; HF, heart failure; NT-proBNP, N-terminal pro-B-type natriuretic peptide; PAD, peripheral artery disease; EF, ejection fraction; 6-MWD, six minute walking test.

Given the results derived from RCTs, international scientific societies currently recommend using GLP1RAs as part of a comprehensive strategy to reduce the risk of CV events in patients with T2DM [21], although they have yet to be recommended to prevent HF [151, 152].

More recently, results of a SELECT study demonstrated the superiority of semaglutide when added to a typical standard of care, compared with placebo, in overweight or obese patients without T2DM, in reducing the incidence of death from cardiovascular causes, nonfatal myocardial infarction, or nonfatal stroke at a mean follow-up of 39.8 months [153]. Semaglutide also showed a non-significant reduction in the heart failure composite endpoint (HR 0.82 [0.71–0.96]).

New molecules are currently being developed and tested. For example, tirzepatide is a dual agonist of glucose-dependent insulinotropic polypeptide and GLP1R, thereby constituting a novel treatment option for T2DM. This agent exerts additional effects in addition to improvement in glycemic control, which can benefit individuals with T2DM, especially those at risk for or with established CV disease or HF. However, current evidence is limited, although it is suggestive of the cardiovascular safety of tirzepatide [150]. SUMMIT is an ongoing RCT that will assess the efficacy and safety of tirzepatide (LY3298176), compared with the placebo, in participants with the obesity phenotype of HFpEF [154, 155, 156, 157, 158, 159, 160, 161, 162].

## 12. Potential Effects of Vericiguat in HFpEF 

Vericiguat is a drug that stimulates the cGMP pathway through direct and indirect stimulation of soluble guanylate cyclase. The current mechanism increases nitric oxide synthase (eNOS) at the endothelial level, directly affecting cardiac workload by reducing systemic resistance and improving vascular compliance [156, 157]. The NO availability enhancement leads to smooth muscle cell relaxation and reduces hypertrophy, inflammation, and fibrosis [158]. Two recent trials demonstrated a potential drug effect in HFpEF: The SOCRATES-PRESERVED study showed no significant effect on mortality and hospitalization in patients with worsening heart failure. However, using vericiguat was associated with better tolerance and quality of life after a 3-month follow-up period [159]. More recently, the VITALITY study did not confirm preliminary findings, showing no differences in quality of life and 6-minute walking distance score [160]. These contrasting data could depend on the high frailty burden of enrolled patients and the short observational period [161].

## 13. Future Perspectives

Even though recent *post-hoc* analyses revealed that all drugs endorsed for patients with reduced ejection fraction have positive effects in subjects with HFpEF up to the cutoff <60%, there are still doubts regarding the effective benefits of all agents [162]. Presently, only SGLT2i have demonstrated a comparable effect in HFrEF and HFpEF, but the remaining data are based on putative analyses, surrogate endpoints, and retrospective data. Since HFpEF encompasses several subtypes with different risk profiles, patients’ frailty, pathophysiological mechanisms, and hemodynamic cardiovascular disorders, a tailored management algorithm needs to be precise according to these features. In particular, there are two settings in which therapeutic advances demonstrated significant improvement: inflammatory and metabolic pathways can be regulated by treatment, resulting in improved functional status, reduced HF hospitalization, and lower CV mortality.

## 14. Conclusions

In the last 5 years, additional putative analyses and *post-hoc* investigations in certain HFpEF phenotypes have indicated that traditional treatment used for HFrEF can be extended to individuals affected by HFpEF. The efficacy of various treatments helped to understand the pathophysiological mechanisms behind the development and maintenance of the HFpEF condition, resulting in the identification of new therapeutic targets. However, more research is needed to understand how these agents influence the natural history of specific HFpEF phenotypes. This is extremely important given the high frequency and poor prognosis of patients with HFpEF.
